# Single-molecule RNA-FISH analysis reveals stochasticity in reactivation of latent HIV-1 regulated by Nuclear Orphan Receptors NR4A and cMYC

**DOI:** 10.21203/rs.3.rs-4166090/v1

**Published:** 2024-04-19

**Authors:** Annalena LaPorte, Rajiv Pathak, Carolina Eliscovich, Laura Martins, Rachel Nell, Adam Spivak, Masako Suzuki, Vicente Planelles, Robert Singer, Ganjam Kalpana

**Affiliations:** Albert Einstein College of Medicine; Albert Einstein College of Medicine; Albert Einstein College of Medicine; University of Utah School of Medicine; University of Utah School of Medicine; University of Utah; Albert Einstein College of Medicine; University of Utah; Albert Einstein College of Medicine; Albert Einstein College of Medicine

## Abstract

HIV-1 eradication strategies require complete reactivation of HIV-1 latent cells by Latency Reversing Agents (LRA). Current methods lack effectiveness due to incomplete proviral reactivation. We employed a single-molecule RNA-FISH (smRNA-FISH) and FISH-Quant analysis and found that proviral reactivation is highly variable from cell-to-cell, stochastic, and occurs in bursts and waves, with different kinetics in response to diverse LRAs. Approximately 1–5% of latent cells exhibited stochastic reactivation without LRAs. Through single-cell RNA-seq analysis, we identified NR4A3 and cMYC as extrinsic factors associated with stochastic HIV-1 reactivation. Concomitant with HIV-1 reactivation cMYC was downregulated and NR4A3 was upregulated in both latent cell lines and primary CD4^+^ T-cells from aviremic patients. By inhibiting cMYC using SN-38, an active metabolite of irinotecan, we induced NR4A3 and HIV-1 expression. Our results suggest that inherent stochasticity in proviral reactivation contributes to cell-to-cell variability, which could potentially be modulated by drugs targeting cMYC and NR4A3.

## Introduction

Efforts to eradicate HIV-1 are hampered by the presence of long-lived, transcriptionally silent latent reservoirs^1-3^. In patients on continuous suppressive therapy, occasional ‘blips’ in plasma viremia suggest the reactivation of latent reservoirs by unknown stimuli^4^. ‘Shock and kill’ therapy aims to eliminate HIV reservoirs by activating latent provirus using ‘Latency Reversing Agents’ (LRAs) and targeting the producer cells for killing^2,5^. Early studies to induce proviral activation in patients using HDAC inhibitors were unsuccessful^6^. A subsequent study revealed that only a tiny fraction of the HIV reservoir (~ 1/60th of replication-competent proviruses) produced infectious progeny virions^7^. The reason for this low and variable level of activation has been attributed to cellular/viral heterogeneity including differences in epigenetic regulation, sites of integration, viral mutations, cellular factors, and/or transcriptional/post-transcriptional blocks^2^. Similarly, cell culture and *ex vivo* models of latency showed reactivation of a small fraction of proviruses^8^. One reason for the observed variability in reactivation could be the inherent stochasticity or ‘noise’ in transcriptional activation, which is well known in prokaryotic as well as eukaryotic gene expression but not well understood during reactivation of latent provirus.

The eukaryotic transcription is characterized as “bursty” or “stochastic” as opposed to “Poissonian”^9-13^. The transcriptional bursting can exhibit different burst size (related to the number of RNA polymerases engaged in the transcription), frequency and duration during active phase of transcription. The bursting parameters can be quantified by the number and intensity of transcription sites (TS) in the population of cells. The transcriptional “noise” increases as the magnitude of transcription or “burst size” increases^9-11^. The stochasticity is controlled by both intrinsic and extrinsic causes^14-16^. ‘Intrinsic noise’ is due to *cis*-elements and fluctuations of *cis*-factors at the promoter site and the ‘extrinsic noise’ is the phenotypic cell-to-cell variations in the levels or activity of factors required for gene expression. Several studies that analyzed stochasticity in HIV-1 expression using LTR-GFP-IRES-Tat reporters indicated that cell-to-cell fluctuations in Tat levels influence the establishment and reactivation of latent provirus^17^. Additional studies have established that the mutations in the transcription factor binding sites Sp1 and NF-kB, integration site and other mutations in viral genome account for variability in proviral transcription^18,19^. However, in these studies, stochasticity was determined by measuring a reporter rather than directly quantitating HIV-1 transcripts. Furthermore, while these studies implicated several intrinsic factors that influence stochastic reactivation, the extrinsic factors that account for cell-to-cell variability were not explored.

To directly measure the cell-to-cell variability in transcription of latent provirus, we applied a method termed SMIRA (Single cell and single Molecule Immunofluorescence and quantitative RNA-FISH Analysis) to analyze the Tat-dependent and -independent reactivation of full-length HIV-1 provirus using cell line models of latency. Our method combines: i) Stellaris-based single molecule RNA fluorescence in situ hybridization (smRNA-FISH); ii) quantitation of unspliced HIV-1 transcripts using FISH-Quant, a spot-detection algorithm customized in MatLab software^26^ ; iii) immunofluorescence (IF) and iv) analysis of thousands of cell images on a slide using High Speed and High-Resolution Scanning (HSHRS). Our studies revealed that reactivation of latent provirus is stochastic both in the presence and the absence of LRAs. Furthermore, single-cell RNA-seq (scRNA-seq) analysis of uninduced cells identified extrinsic factors associated with stochastic reactivation of latent provirus. Finally, we demonstrate that a drug that regulates the expression of extrinsic factors of stochasticity can influence LRA-mediated reactivation of HIV-1.

## Results

### A quantitative temporal analysis of HIV-1 reactivation in latent cells

**Figure 1:**
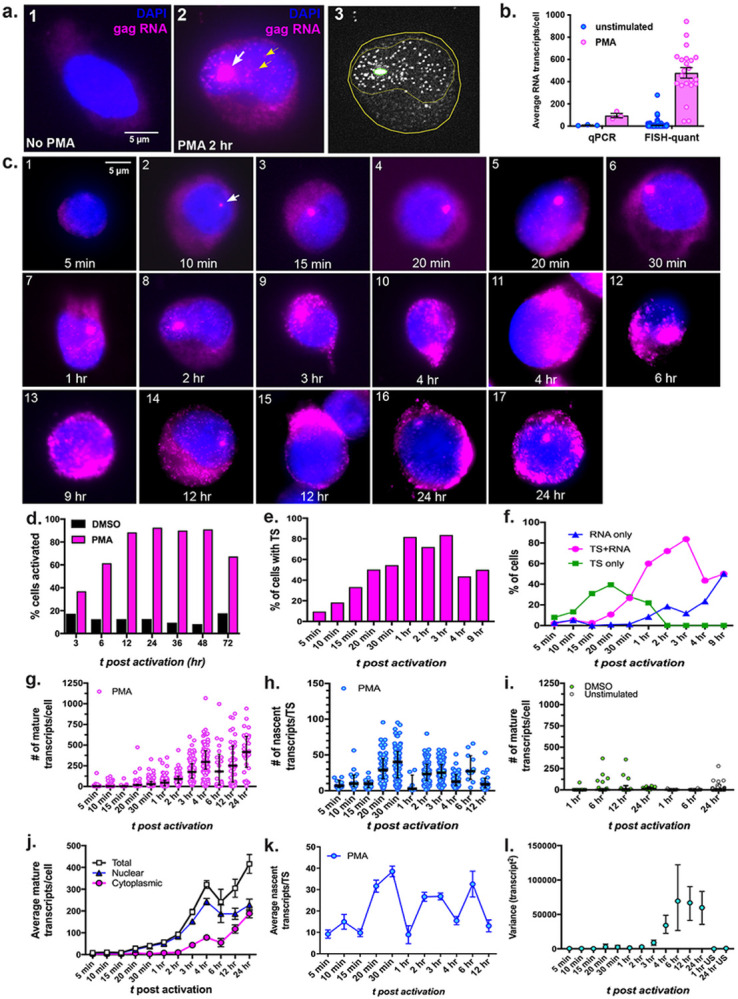
Quantitative smRNA-FISH analysis to determine the reactivation kinetics of latent proviruses: (**a**) Establishment of quantitative smRNA-FISH to study proviral transcription. Representative images of ACH2 cells before and after latency reversal. Panel 1, no stimulation, panel 2, stimulation with PMA. Magenta color represents HIV-1 unspliced gag RNA. The white arrow in the panel 2 points to the TS, and small yellow arrows point to single gag RNA molecules. Panel 3 is the filtered image of the cell from Panel 2 for FISH-quant analysis; (**b**) Comparative analysis of FISH-Quant and qRT-PCR of the same sample; graph represents mean +/− SEM; (**c**) A time course analysis of proviral reactivation, showing the appearance of TS, accumulation of RNA in the nucleus, in the cytoplasm, and in the cell periphery. Each panel shows a representative cell image; (**d-f**) HSHRS analysis of reactivated cells to determine the % of cells containing *gag* RNA only (***d***), TS only (***e***), and both *gag* RNA and TS (***f***); (**g-i)** Result of FISH-quant analysis of transcripts per cell. Representation of # of mature transcripts per cell (***g***) and # of nascent transcripts per TS (***h***) in PMA treated cells, and # of mature transcripts per cell in DMSO treated cells (***i***) at various time points. Each dot represents data from a single cell or single TS, determined by FISH-quant. The graphs are scatter dot plots with median with interquartile range; (**j-k**) Kinetic analysis of reactivation represented as average number of transcripts present per cell (total, data are derived from the graph ***g***), per nucleus or cytoplasm (***j***); and average number of nascent transcripts per TS, derived from the graph ***h (k).*** The graphs represent mean +/− SEM; (**I**) Variance analysis (standard deviation^2^) indicates the high degree of variability in the number of transcripts/cell.

The ACH2 latent cells harbor a full-length HIV-1 provirus, which is non-responsive to Tat due to a point mutation in TAR^20-22^. Since Tat fluctuations account for stochasticity in HIV-1 transcription, we used these cells to identify factors other than Tat that influence stochastic reactivation of latent provirus. To quantify un-spliced full-length HIV-1 RNA at single molecule level in reactivated cells, we employed 40 tandem 21-mer oligonucleotide probes (Stellaris^®^) corresponding to contiguous sequences in *gag*, each with a fluorescent label (Fig. 1 and Supplementary Table ST1). Exposure of ACH2 to PMA for as little as 2h led to a burst of transcription from the provirus, which was visible as a single large bright spot in the nucleus corresponding to a transcription site (TS) that marks the site of proviral integration (Fig. 1a, panel 2, white arrow)^23-25^. Single molecules of RNA were detected as diffraction-limited spots in the nucleus (Fig. 1a, panel 2, yellow arrow heads). We employed FISH-quant to quantify the individual gag RNA molecules within a cell and within the TS (Fig. 1a, Panel 3)^26^. A comparative analysis of an average number of RNA molecules/cell obtained by FISH-quant vs. qRT-PCR of the same sample indicated that RNA-FISH-Quant analysis is more sensitive in predicting HIV-1 RNA molecules/cell, likely owing to the minimal sample processing involved in this method (Fig. 1b).

A temporal analysis of reactivation was conducted by inducing ACH2 cells with PMA for various time points and subjecting them to smRNA-FISH. Images from 50–200 cells were captured for each time point. Distinct TSs appeared in the nucleus as early as 10 min. post reactivation (p.r.) (Fig. 1c, panel 2, white arrow), the size of which increased over time (Fig. 1c panels 2–6). The individual mature mRNAs were visible by 15–30 minutes p.r. as punctate spots within the nucleus, which appeared to move away from the TS (Fig. 1c, panels 3–5). RNA started appearing in the cytoplasm by ~ 3 h p.r. (Fig. 1c, panel 9). By 12–24 h accumulation of RNA could be seen in the cytoplasm as a bright cap (Fig. 1c, panel 15 and 16). HSHRS analysis of > 10,000 cells per time point indicated that by about 12 h p.r. the maximum number of cells (~ 80%) were activated and were positive for either gag RNA and/or TS (Fig. 1d). The percentage of cells with TS increased over time and by about 1–3 h p.r. 60–80% ACH2 cells harbored distinct TSs (Fig. 1e). After 3 h p.r., the number of cells with TS decreased and many cells at this point harbored mRNAs but no TS (Fig. 1f). These results indicated that reactivation from latent provirus is rapid, occurs within ~ 20 min. p.r. and that the first round is completed within a span of 3–4 hours, as, at this time, many cells harbor RNA but no TS (Fig. 1f).

### Stochasticity in HIV-1 reactivation in ACH2 cells

We determined the absolute numbers of mature transcripts per cell and transcripts per TS (burst size), by evaluating 10–50 cells per time points using FISH-Quant (Fig. 1g-l). The total number of mature transcripts/cell increased steadily but was highly variable from cell-to-cell. As the magnitude of transcription increased, the variability also increased with a range from 0-1000 transcripts/cell (Fig. 1g, note that each dot in the graph represents the total number of mature transcripts per single cell). Quantitation of nascent *gag* RNA per TS indicated that the burst size varied highly from cell to cell at any given time, and that a maximum of ~ 100 nascent transcripts/TS were detected (Fig. 1h). Quantitative analysis of subcellular distribution of mature *gag*RNA indicated that until ~ 2 h p.r., most transcripts were present within the nucleus and the cytoplasmic accumulation was observed starting from ~ 3 h p.r. (Fig. 1c and 1j). The average number of nascent RNA/TS steadily increased, and several peaks of transcripts were observed indicating that the transcription initiation proceeded in waves (Fig. 1k). These results indicated that the HIV-1 reactivation is intrinsically stochastic or “bursty” and that the transcription proceeded in pulses/waves after stimulation.

To quantitate the degree “noise” in transcription activity, at a given time point, we calculated the Fano factor, a statistical method to determine the stochasticity, by determining the ratio between the cell-to-cell variation in the number of transcripts and the mean of the transcripts/cell. Variance analysis indicated that the number of transcripts/cell fluctuated more in the later time points than in the early time points (Fig. 1l and Supplementary Fig. S1a). However, Fano factor was greater than 1 at all-time points measured, indicating an inherent burstiness of transcription (Supplementary Fig. S1b). These studies indicated that there is stochastic activation of HIV-1, even when it is Tat-independent.

We subjected two additional latent T-cell lines, J1.1 and J-Lat, to smRNA-FISH analysis followed by HSHRS (Supplementary Fig. S2). In these cells TS were present, and the reactivation occurred rapidly. A maximum of 80% and 20% cells were reactivated in J-Lat and J1.1, respectively (Supplemental Fig. S2). J1.1 cells exhibited fluctuations in transcription with waves every few hours (Supplemental Fig. S2d).

### Time course analysis of Gag protein synthesis

We studied the kinetics of expression of Gag protein, by combining smRNA-FISH with IF using α-p24 antibody (Supplementary Fig. S3). Gag protein was detected at 6 h p.r. at the nuclear periphery, by ~ 12 h, diffusely in the cytoplasm on one side, and by 24 h diffused throughout the cytoplasm and plasma membrane (Supplementary Fig. S3a). In some cells, gag-RNA and proteins co-localized in extreme quantities on one side of the cytoplasm, forming a cap-like structure (Supplementary Fig.S3a, panels 17–20). HSHRS analysis indicated that maximum cells that express RNA and/or Gag protein were detected at ~ 24 h p.r. which decreased at 48 and 72 h p.r. (Supplementary Fig.S3b). In DMSO controls, the positive cells remained at ~ 5%, 24–72 h p.r. (Supplementary Fig. S3c).

### Effect of LRAs on the reactivation kinetics of latent provirus

**Figure 2:**
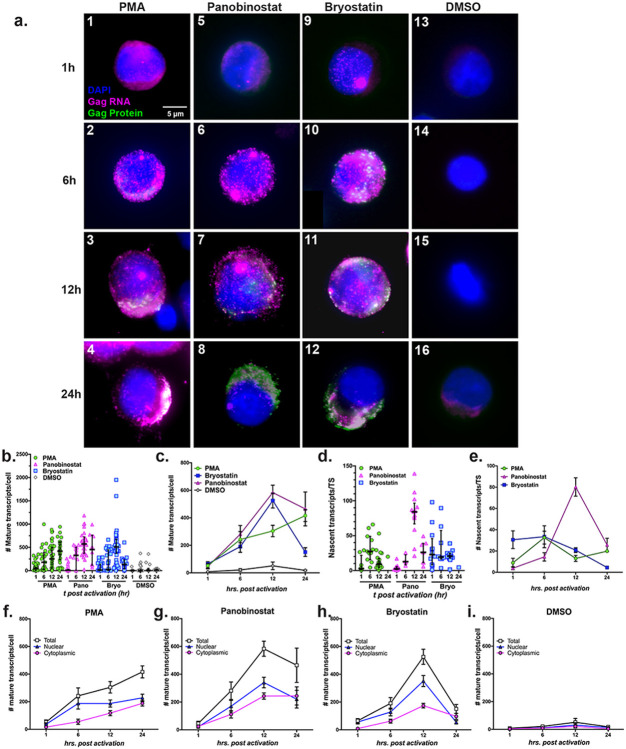
Stochastic reactivation of HIV-1 by LRAs in ACH2 cells: (**a**) A time course analysis to determine the kinetics of reactivation by DMSO (control), LRAs PMA, panobinostat and bryostatin at 1, 6, 12 and 24 h p.r. Each panel shows a representative cell image after the application of combination of smRNA-FISH+IF using a-p24 antibody. Blue= DAPI, Magenta =gag RNA, Green = p24, and White = overlay of gag RNA and p24. TS appears as bright magenta colored spot within the nucleus. (**b-i**) FISH-quant analysis of reactivation by LRAs; (**b and c**) Number of transcripts per cell, represented as scatter plot from individual cells (***b***), and as average from many cells, derived from ***b*** (***c***); (**d and e**) Number of nascent transcripts per TS, represented as scatter plot from individual TS (***d***), and as average from many TS, derived from ***d*** (***e***); (**f-i**) Subcellular distribution of transcripts upon reactivation by LRAs. The graphs represent average transcripts present in nucleus, cytoplasm and total in cells treated with individual LRAs as indicated. Scatter dot plots (***b*** and ***d***) are overlayed with median with interquartile range, and all line graphs (***c, e, f-i***) represent mean −/+ SEM.

To determine if clinically relevant LRAs lead to stochastic reactivation, we examined the effect of PKC modulator bryostatin and HDAC inhibitor panobinostat, in addition to PMA on the reactivation kinetics in ACH2 cells (Fig. 2a). The drugs exhibited three distinct kinetics of reactivation (Fig. 2b-e). First, bryostatin and panobinostat exhibited rapid reactivation kinetics with a peak at 12 h p.r. (Fig. 2b-c). By 24 h the bryostatin-induced reactivation declined rapidly as compared to PMA and panobinostat (Fig. 2b-c). Bryostatin induced larger TS (containing up to 98 nascent trasncripts/TS) at very early time points at 1 h p.r. indicating a swift transcription initiation when compared to that of PMA (up to 29 trasncripts/TS) and panobinostat (up to 17 transcripts/TS), at the same time point (Fig. 2a, d and e). On the contrary, panobinostat showed peak activity in transcription initiation at 12 h p.r., which was also correlated to an increase in the number of cells expressing large TS and the number of nascent transcripts/TS (Fig. 2a, panel 7 and Fig. 2d and e). In summary, while bryostatin showed rapid initiation of transcription that steadily declined, panobinostat showed a slow initiation that peaked at 12 h p.r., which declined rapidly (Fig. 2d and e). The proviral reactivation was stochastic in the presence of each of the three drugs as indicated by the variability in the mature transcripts/cell and the burst size (Fig. 2b and d). Analysis of kinetics of cytoplasmic accumulation indicated no blocks in this process with these drugs (Fig. 2f-i).

### A combination of panobinostat and bryostatin facilitates sustained viral transcription:

**Figure 3:**
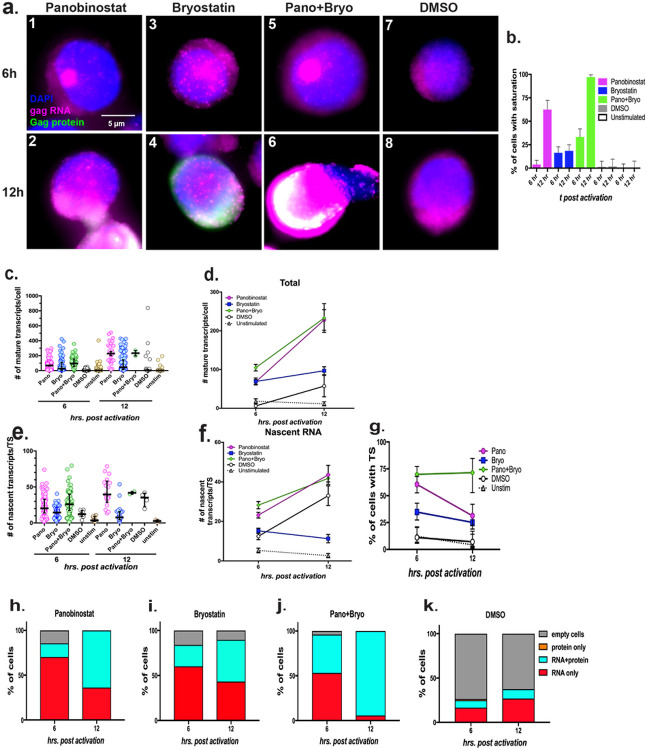
Analysis of reactivation of HIV-1 by combination of panobinostat and bryostatin: (**a**) A time course analysis to determine the kinetics of reactivation by combination of LRAs panobinostat and bryostatin at 6 and 12 h p.r. DMSO was used as a control. Each panel shows a representative cell image after smRNA-FISH+IF using a-p24 antibody. Blue = DAPI, Magenta =gag RNA, Green = p24, and White = overlay of **gag**RNA and p24. TS appear as bright magenta colored spots within the nucleus; (**b**) Graphical representation of the percentage of cells with saturated **gag**RNA expression upon LRA treatment; (**c-g**) Quantitate reactivation kinetics by combination of LRAs using FISH-quant analysis. (**c and d**) Number of transcripts per cell, represented as scatter plot from individual cells, meadian with interquartile range (***c***), and as average from many cells, mean +/− SEM (***d***). (**e and f**) Number of nascent transcripts per TS, represented as scatter plot from individual TS, median with interquartile range (***e***), and as average from many TS, mean +/− SEM (***f***). (**g**) Percentage of cells that contain TS at 6 and 12 h p.r. using single and combination LRA, as determined by HSHRS. Graphs represents mean −/+ SEM. (**h-k**) Graphic representation of percentage of cells expressing gag RNA or p24 protein or both in response to single and combination LRAs, as obtained from HSHRS analysis.

Since the LRAs exhibited different kinetics of reactivation, we surmised that a combination of drugs may lead to better activation. We tested reactivation by combining panobinostat and bryostatin, at 6 and 12 h p.r., (Fig. 3). Combination treatment resulted in an increased number of cells that over-expressed RNA by 12 h p.r. such that the images from these cells were saturated and could not be included for FISH-Quant analysis (Fig. 3a, panel 6; and Fig. 3b). With this limitation, the analysis of the remaining cells indicated that the combination treatment resulted in a similar number of mature transcripts/cell and burst size to that of panobinostat treatment at 6 h p.r. (Fig. 3c-f). However, the combination drug treatment resulted in a sustained high percentage of TS harboring cells at 12 h compared to that of 6 h, whereas single drug treatment exhibited a decrease in percentage of TS containing cells at 12 h p.r. (Fig. 3g). Thus, the combination drug treatment resulted in a sustained activation of transcription (Fig. 3g). Consistent with these observations, close to 100% of the cells were reactivated with combination drug treatment (Fig. 3h-k).

### Analysis of reactivation in primary latent CD4^+^ T cells

To study the reactivation kinetics in primary cells, we applied RNA-FISH and HSHRS to an *ex vivo* latency model^29^. Analysis of primary CD4^+^ latent T-cells, reactivated using α-CD3/CD28 antibodies, indicated the clear presence of *gag*-RNA, TS, and Gag proteins (Supplementary Fig. S4a, panels 1–16). Analysis of % of cells positive for RNA, protein or both indicated that while % of protein positive cells increased, those containing RNA alone decreased suggesting that the peak of transcriptional reactivation occurred 6 h p.r. or earlier (Supplementary Fig. S4b). Interestingly, even the uninduced controls exhibited the presence of low % of reactivated cells (Supplementary Fig. S4c, and S4a, panels 17–24). Most of the cells expressing HIV-1 RNA were not amenable for FISH-quant analysis due to near saturating amounts of RNA in these cells.

### Singles-cell RNA seq to determine the factors responsible for stochastic activation

We observed transcriptional ‘noise’ even in uninduced ACH2 cells, and in primary cells (Supplementary Fig. S4c), consistent with stochastic activation. About 5–20% of the uninduced ACH2 cells were activated at any given time point, but with a smaller number of mature transcripts/cell (maximum of ~ 250 transcripts/cell, Fig. 1d, 1i, and Supplementary Fig. S5 and Supplementary Table ST2). Thus, the basal level expression reported previously as “leaky expression” of provirus using the pooled samples in uninduced conditions, is likely due to stochastic activation in some cells^21^. Furthermore, 2–10% of J-Lat, J1.1, and OM10.1 cells were also reactivated under uninduced conditions (Supplementary Fig. S5 and Supplementary Table ST2).

**Figure 4:**
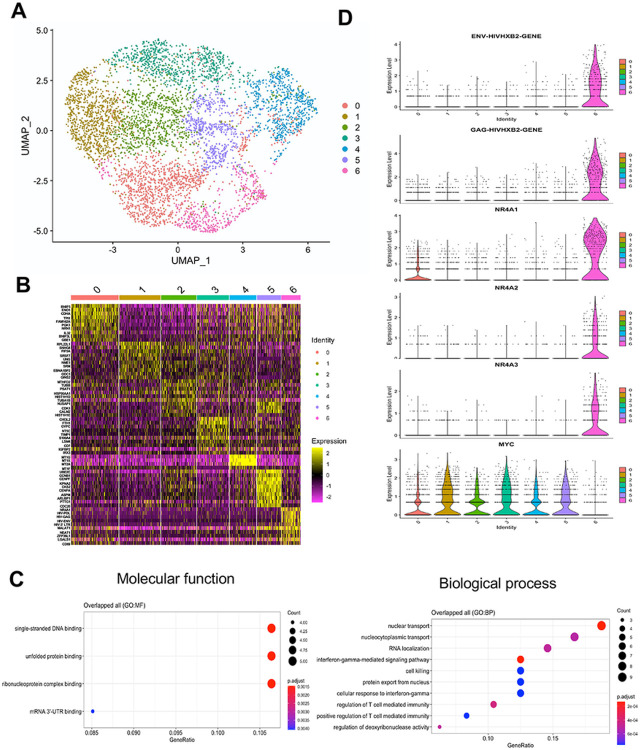
scRNA-seq analysis of uninduced ACH2 cells showing the presence of a distinct group of cells positive for HIV-1 transcripts. **(a)** UMAP plot analysis to indicate seven distinct clusters in latent ACH2 cells, out of which cluster 6 is positive for HIV-1 transcripts. In the cluster, each dot corresponds to a single cell; **(b)** Heat map analysis of the top 10 upregulated genes in each cluster; **(c)** Gene ontology (GO) enrichment analysis represents the genes in two independent categories, molecular function, and biological process. The dot size represents the count of overlapped genes whereas the gene ratio represents the count of overlapped genes for each GO item divided by the total number of genes; **(d)** Violin plot to indicate the distribution and expression levels of HIV-1 transcripts (Env and Gag gene), NR4A1, NR4A2, NR4A3, and cMYC in each of the seven distinct clusters.

Stochastic activation is regulated both by intrinsic and extrinsic factors, where the ‘extrinsic noise’ is the phenotypic cell-to-cell variations in the levels or activity of factors required for gene expression^14-16^. To determine the reason for stochastic activation of cells under uninduced conditions, we performed single-cell RNA-seq (scRNA-seq) analysis to determine differences in transcriptome of reactivated and unreactivated cells within the same pool using 10x Genomics. The resulting UMAP data of ~ 5,000 ACH2 cells indicated the presence of 7 closely related clusters, with cluster 6 showing high-level expression of HIV-1 Gag, Pol and Env (Fig. 4a-d). Heatmap analysis indicated that HIV-1 genes were among the top 10 highly upregulated genes in cluster 6 (Fig. 4b and Supplementary Table ST3). Gene ontology (GO) analysis indicated that single-stranded DNA binding, unfolded protein binding and ribonucleoprotein complex binding are the three top molecular functions, and nuclear transport, nucleocytoplasmic transport and RNA localization are the top biological functions in the differentially expressed genes (DEGs) within cluster 6 (Fig. 4c). Consistent with the GO analysis, several lncRNA-encoding genes, including MALAT1 and NEAT1, which had been reported to influence HIV-1 infection and reactivation, were part of the top ten upregulated genes in cluster 6, validating our data (Fig. 4b and Supplementary Table ST3). MALAT1 is a lncRNA that reactivates HIV-1 by replacing the polycomb repressive complex from HIV-1 LTR^27^. NEAT1 is upregulated during HIV-1 replication and influences export of HIV-1 RNA from the nucleus^28,29^. LGALS1 or Galectin-1 is another top gene in the group that has been shown to enhance HIV-1 replication^30^. HIV-1 expression was not influenced by cell cycle stages, as cells at all different stages of cell cycle were represented in cluster 6 (Supplementary Fig. S6a and b).

### NR4A overexpression was correlated to stochastic activation of HIV-1

**Figure 5:**
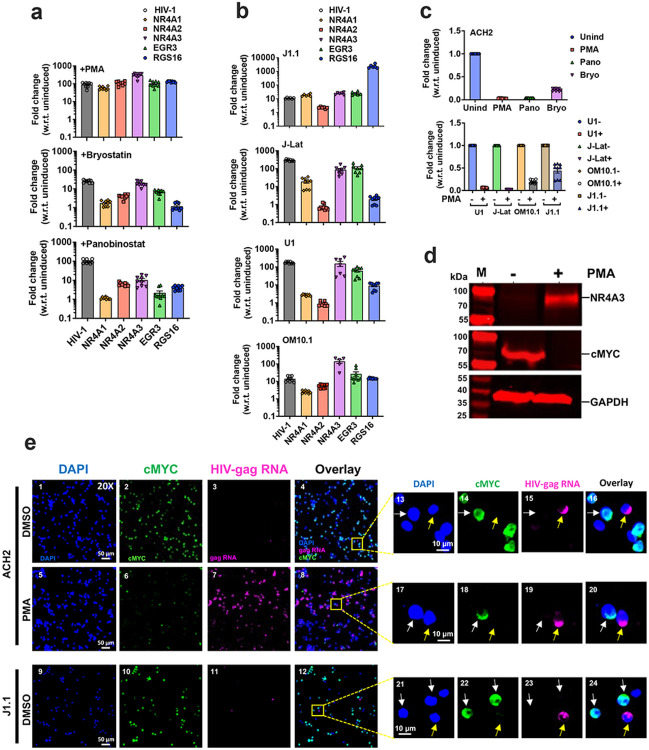
Validation of scRNA-seq results by qRT-PCR, smRNA-FISH+IF and Western analysis, under induced and/or uninduced conditions. **(a)** The activation of differentially expressed genes (DEGs) identified in stochastically activated cells in response to various LRAs PMA (top panel), bryostatin (middle panel) and Panobinostat (bottom panel), in pools of ACH2 cells; **(b)** qRT-PCR data showing the differential expression of key genes identified in stochastically activated ACH2 cells in various latent cells including J1.1 (top panel), J-Lat (second from the top panel), U1 (third from the top panel), and OM10.1 (bottom panel) in response to PMA; **(c)**qRT-PCR analysis of the downregulation of cMYC gene in response to various LRA treatments (PMA, bryostatin, panobinostat, respectively) along with uninduced (Unind) conditions in ACH2 cells (top panel), and qRT-PCR analysis of the downregulation of cMYC in response to PMA treatments in various latent cells, including J1.1, J-Lat, U1, and OM10.1 cells (lower panel); **(d)** Western blot analysis showing upregulation of NR4A3 and downregulation of cMYC in reactivated HIV-1 latent ACH2 cells at the protein level; **(e)** RNA-FISH and IF showing the expression of cMYC and HIV-1 RNA at single cell level in uninduced (DMSO) and PMA-induced ACH2 and J1.1 cells (left panel), and magnified areas of the same images (right panels) demonstrating the inverse correlation of expression of cMYC and HIV-1 RNA at the single-cell level. Graphs represents mean +/− SEM.

The HIV-1 containing cluster 6 formed a branch that was distinct from the rest of the clusters, based on ClusterTree analysis (Supplementary Fig. S6c). The characteristic genes of node 8 that separated cluster 6 from rest of the clusters identified HIV-1 Gag, Pol, Env, MALAT1, NEAT1, and three nuclear orphan receptor genes belonging to the family 4A, NR4A1, 2, and 3 (Supplementary Table ST4). Violin plot analysis indicated that all three NR4A genes are differentially upregulated in cluster 6 (Fig. 4d). To validate the association of cluster 6 genes with activation of HIV-1, even in the induced conditions, we carried out qRT-PCR analysis of RNA isolated from pools of induced latent cells (Fig. 5). We found that PMA upregulated all the genes from the cluster 6 tested along with HIV-1 RNA (Fig. 5a, top panel). Bryostatin and panobinostat treatment upregulated these genes to variable degrees (Fig. 5a, middle and bottom panels). Treatment of other latency models such as J1.1, J-Lat, U1 and OM10.1^21,31,32^ with PMA resulted in upregulation of most but not all the genes (Fig. 5b). Interestingly NR4A3 was consistently upregulated in all the reactivated latent cell lines and across the three different LRAs (PMA, bryostatin and panobinostat), and correlated to upregulation of HIV-1, suggesting that NR4A3 could be a common factor that determined the stochastic upregulation of HIV-1.

NR4A1 (Nur77), NR4A2 (Nurr1), and NR4A3 (Nor1) are closely related proteins belonging to the orphan nuclear receptor family^33^. These proteins were initially defined as nerve growth factor induced receptors that bind to NGFI-β-response element^33^. NR4A receptors bind as homo- or hetero-dimers with each other to Nurr-responsive element^33^. NR4A1 and 2 can also dimerize with retinoid X receptor to bind to a D5 motif, and NR4A1 is a co-factor for Sp1-regulated genes^33^. Despite the similarities between NR4A1, 2, and 3, and their similar ability to bind to common *cis*-regulatory elements, these proteins exhibit unique activities and cell/tissue-specific functions. It is interesting to note that NR4A2 directly binds and recruits CoREST complex to HIV-1 promoter to repress HIV-1 transcription in microglia^34^. Our results indicated that NR4A2 was either repressed or not highly activated in various cell lines during HIV-1 reactivation, consistent with this observation.

### cMYC is inversely correlated to stochastic reactivation of HIV-1 provirus

*NR4A1 and 3* share overlapping functions and loss of these genes leads to acute myeloid leukemia (AML) in mice, and thus are considered as tumor suppressors^33,35^. Furthermore, it has been demonstrated that cMYC is the target of NR4A-mediated repression and tumor suppression^36^. Interestingly, we noted that cMYC was one of the downregulated genes in cluster 6 of ACH2 cells, suggesting that upregulation of NR4A genes could lead to downmodulation of cMYC expression (Supplementary Tables ST3 and ST4). In addition, cMYC has been implicated in mediating HIV-1 latency by binding to SP1 at the HIV-1 promoter^37^.

To test the correlation of cMYC down-modulation to NR4A upregulation and HIV-1 reactivation, we tested the effect of different LRAs in ACH2 and other latent cell lines, on expression of cMYC by using qRT-PCR. cMYC was downregulated in ACH2 cells when induced with PMA, bryostatin or panobinostat (Fig. 5c, top panel). Furthermore, cMYC downregulation was correlated to HIV-1 reactivation in all other cell line models tested (U1, J-Lat, OM10.1, and J1.1), (Fig. 5c bottom panel). Furthermore, the expression of NR4A3 and cMYC proteins were inversely correlated and were induced or repressed respectively upon treatment of ACH2 cells with PMA (Fig. 5d). These results are consistent with previous reports of cMYC in inducing latency and provided additional light on the role of cMYC and NR4A3 in stochastic reactivation of HIV-1 latent cells^37^.

To discern that cMYC expression was inversely correlated with HIV-1 expression at single cell level, we performed RNA-FISH + IF analysis of uninduced ACH2 and J1.1 cells using HIV-1 gag RNA FISH probes and α-cMYC antibody (Fig. 5e). We found that cMYC was expressed in a majority of the cells under uninduced conditions in both ACH2 and J1.1 cells, and a few cells were positive for HIV-1 RNA (Fig. 5e, top and bottom row of panels 1–4 and 9–12). On the contrary, PMA treatment induced HIV-1 RNA in a majority of ACH2 cells and only a few cells were positive for cMYC (Fig. 5e, middle panels 5–8). Furthermore, within the majority of single cells, in both uninduced and induced conditions, cMYC and HIV-1 RNA expression were inversely correlated (Fig. 5e, panels 13–24 white and yellow arrows point to cMYC and HIV-1 RNA, respectively). These results are consistent with stochastic reactivation of HIV-1 in the absence of cMYC at single cell level.

To determine if cMYC was a common factor for regulating HIV-1 reactivation in all the cell lines, we carried out scRNA-seq analysis of two additional cell lines J1.1 and J-Lat under uninduced conditions. About 5,000–10,000 cells were subjected to scRNA-seq and the data from these two sets of cell lines were merged with that of ACH2 to identify a common pool of cells expressing HIV-1. UMAP analysis indicated that even when combined, the HIV-1 expressed cells clustered separately from the rest of the cells (Supplementary Fig. S7). There were 12 clusters from the combined analysis (Supplementary Fig. S7a). The top 10 genes in cluster 11 were HIV-1 genes, as indicated in the heat map analysis (Supplementary Fig. S7b and Supplementary Table ST5). Further analysis indicated that most of DEGs commonly identified in the latent cell lines were repressed targets (Supplementary Table ST5) and DEGs in cluster 11 formed a network containing cMYC, and the genes associated with its function such as EGR1, CEBPE, NPM1, TOP2A, KPNA2, highlighting the importance of cMYC pathway in regulating the expression of HIV-1^38-43^ (Supplementary Fig. S7c).

### Upregulation of NR4A3 and downregulation of cMYC in patient-derived samples

**Figure 6:**
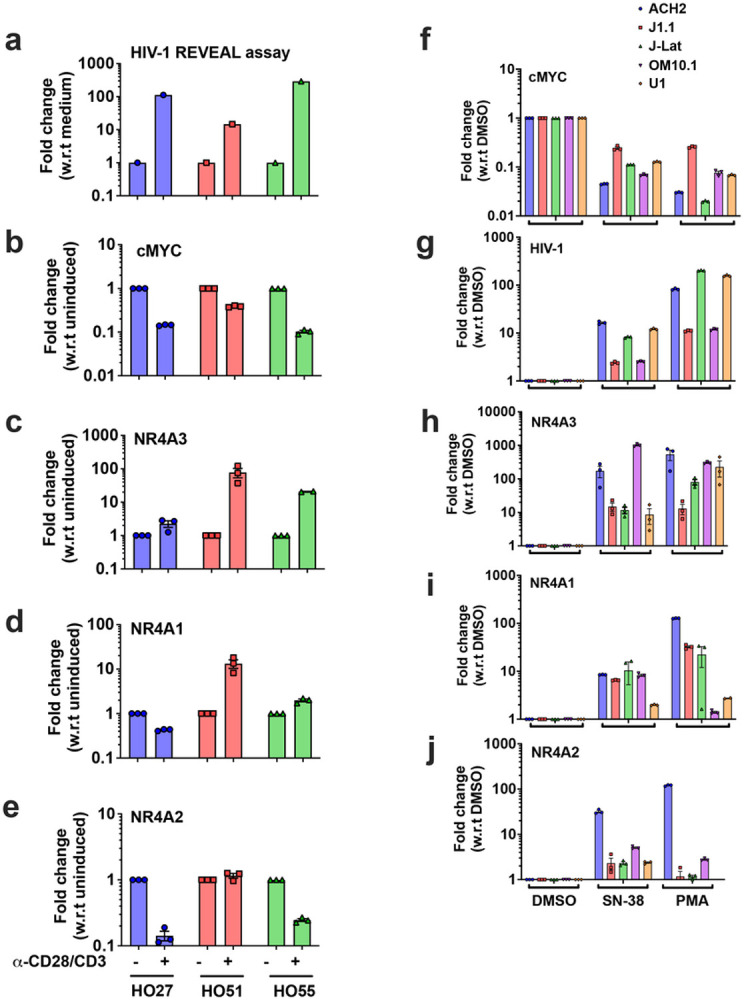
Correlation of NR4A and cMYC expression to HIV-1 reactivation in patient samples and effect of SN-S8 on the expression of NR4A, cMYC and HIV-1 in various latent cell lines. **(a)** REVEAL assay confirming the activation of HIV-1 by α-CD28/CD3 Ab in three different primary CD4^+^ T-cells derived from aviremic patients (HO27, HO51, and HO55); qRT-PCR data showing the downregulation of cMYC (***b***) and upregulation of NR4A3,(***c***) NR4A1 *(**d**)*, and NR4A2 *(**e**)*, in each of the three patient samples; (**f-j**) SN-38, an inhibitor of cMYC reactivates HIV-1 and is correlated to the upregulation of NR4A3 and downregulation of cMYC in ACH2, J1.1, J-Lat, OM10.1, and U1 cells. qRT-PCR data showing the downregulation of cMYC (***f***), and upregulation of HIV-1 (***g***), NR4A3 (***h***), NR4A1 (***i***) and NR4A2 (*j*), in response to SN-38 in 5 latent cell lines. PMA was used as a positive control (***f-j***). Graphs represents mean +/− SEM.

We tested to determine if NR4A3 and cMYC expression were correlated to reactivation of HIV-1 in patient derived cells. Latent CD4^+^ T-cells isolated from three independent aviremic HIV-1 patients (HO27, HO51, and HO55) were reactivated by α-CD28/CD3, and RNA was isolated from the virions and the cells. Rapid ex vivo evaluation of anti-latency (REVEAL) assay was used to measure the viral RNA as a way to measure viral particle production^44^, and RNAs isolated from the cells were used to determine the expression of cMYC and NR4A genes via qRT-PCR (Fig. 6a-e). We found that cMYC levels were down-modulated and NR4A3 were up-regulated in induced samples as compared to the uninduced controls in all three patients (Fig. 6b and c). Interestingly, NR4A2, which represses HIV-1 transcription was either down-regulated or unchanged (Fig. 6e). NR4A1 was variably expressed in different samples (Fig. 6d). These results indicated that cMYC and NR4A3 expression were correlated to HIV-1 reactivation in both CD4^+^ T cell lines and in primary CD4^+^ cells isolated from patients.

### NR4A modulator and cMYC inhibitor SN-38 acts as an LRA to reactivate latent HIV-1

NR4A genes activate transcription of cellular genes by binding to common *cis* elements and are induced in response to T-cell activation^45-50^. NR4A genes play an important role in oncogenesis and act as tumor suppressors^36^. In cMYC-dependent AML, small molecule activators of NR4A1 and 3 have been shown to inhibit tumor growth by repressing cMYC^51^. Based on these studies we hypothesized that a drug that targets NR4A and represses cMYC could act as an LRA that can induce activation of HIV-1 provirus. In addition, TOP2A was one of the commonly repressed genes in HIV-1 positive cells, as indicated by network analysis (Supplementary Fig. S7c). We identified SN-38 (7-ethyl-10-hydroxycamptothecin), a derivative of topoisomerase-I inhibitor irinotecan (CPT-11), as one of the drugs that represses cMYC as a part of its mechanism of action^52^. Irinotecan is an FDA approved drug used as an anti-cancer agent and SN-38 is a 100 to 1000-fold more active metabolite of irinotecan^53^. We investigated the effect of SN-38 in inducing HIV-1 in five different latent cell line models, ACH2, U1, J-Lat, OM10.1 and J1.1. Our results indicated that while *cMYC was* repressed, *NR4A3* was upregulated by SN-38 in all the five cell lines (Fig. 6f-h). Consistent with our hypothesis, transcription of HIV-1 was activated in all five cell line models, compared to DMSO treated control (Fig. 6g). Interestingly, the expression of NR4A1 and NR4A2 was much less compared to that of NR4A3 (Fig. 6i and j). Similar results were obtained when the cells were treated with PMA used as a positive control (Fig. 6f-j). These results indicated that SN-38 might act as a novel LRA which can reactivate latent HIV-1 provirus by repressing cMYC and activating NR4A3.

## Discussion

One of the major hurdles in accomplishing an HIV-1 cure is the variability in the latent cell reservoirs. Many factors contribute to this variation. In this report, we have investigated how stochasticity in proviral reactivation contributes to variability. Most studies of HIV-1 reactivation consist of cell population measurements at single time points without quantitation. Use of combination of smRNA-FISH and FISH-quant techniques allow quantitative measurement at a single cell and single molecule level at multiple time points, providing a tantalizing view of the reactivation of latent cells induced by different LRAs. The reactivation in latent T-cells is rapid and stochastic, and it proceeds in waves. Because of the quantitative and sensitive nature of smRNA-FISH, we identified a large variability in mature transcripts ranging from a few to 1000s of transcripts/cell (while the highest previously reported is ~ 300). We were able to determine the progression of transcription initiation by measuring the number of nascent RNA/TS. Use of HSHRS microscopy, allowed the analysis of a large number of cells at a given time point.

The results obtained using our methods provide unique insights into the differences in kinetics of reactivation by LRAs. We found that panobinostat showed a late peak activity in transcription initiation, and bryostatin an early peak of activity. Interestingly, the combination of these LRAs showed an increased reactivation, and nearly 100% of latent cells were reactivated. In the combination drug treatment, the reactivation was sustained for a longer time period than in the single drug treatment, suggesting that monitoring the reactivation kinetics at single cell single molecule level will allow the determination of an effective drug combination for therapy that would induce sustained reactivation in a majority of latent reservoirs. Expanding the window of reactivation time by combination LRAs might provide a greater opportunity to achieve the killing strategy more effectively to eliminate these reservoirs.

We used ACH2 cells to characterize the Tat-independent nature of provirus, since it has been reported that fluctuations in Tat are the cause of cell-to-cell variation^54^. Our study indicates that even in the absence of Tat-mediated regulation, the proviral reactivation is stochastic. We observed stochastic reactivation even in uninduced conditions in all latent cell models tested. It is possible that these stochastic reactivations could be the source of blips of activity observed during long term anti-retroviral therapy (ART) treatment in AIDS patients^55,56^. scRNA-seq analysis of uninduced latent cells identified a group of factors that are selectively associated with HIV-1 reactivation in uninduced conditions. Some of these DEGs are lncRNAs that were reported to influence HIV-1 reactivation in induced conditions, validating our studies and indicating that the same factors are responsible for HIV-1 reactivation in both induced and uninduced conditions. Consistent with this idea, all the factors we identified as associated with stochastically activated HIV-1 expressing cells in uninduced conditions were also correlated to induced reactivation in pooled samples.

Our results indicate that NR4A3 and cMYC expression are correlated to proviral reactivation in latent cell lines and in patient-derived primary latent cells. We hypothesize that NR4A3 represses cMYC expression and a reduced level of cMYC leads to reactivation of HIV-1. It is intriguing to note that MYC protein and MYC locus are implicated in stochastic reactivation of eukaryotic genes and cMYC’s dynamic association with chromatin networks and transcription factors influences stochastic gene expression^57,58^. These studies are consistent with our finding that the levels of cMYC, which can be stochastically controlled, can cause cell-to-cell variability in its expression and on the expression of its downstream effectors such as HIV-1 transcription at the single cell level. Further studies to address the dynamics of cMYC and NR4A3 interactions with HIV-1 and cMYC promoters at single cell and single molecule level are likely to provide further insight into the mechanism of stochastic reactivation of latent provirus. Drugs, such as SN-38, that regulate the NR4A and cMYC network are promising new leads that could be leveraged to reactivate latent cells in HIV cure studies.

## Online Methods

### Cell culture and Drugs for Study:

The latent T-cells ACH2, J1.1, J-Lat, U1, and OM10.1 were obtained from NIH AIDS Reagent program (Cat #s 349, 1340, 9846, 165, and 1319, respectively; RRIDs: CVCL_0138, CVCL_8279, CVCL_8280, CVCL_M769, and CVCL_D567, respectively) and were maintained in RPMI-1640 (HyClone; Cat # SH30096.01) supplemented with 2 mM L-glutamine (Gibco; Cat # 25030-081), 100 U/ml penicillin, 100 μg/ml streptomycin (Gibco; Cat # 15140-122), 10% fetal bovine serum (Atlas Biologicals; Cat # F-0500-A). ACH2 cells were diluted to 10^6^ cells/mL and cultured in RPMI-1640 + 10 mM HEPES (Sigma; Cat # H0887). Cells were activated at 2x10^5^ cells/mL. HIV-1 was induced by the addition of the following drugs, 30 nM panobinostat (Cat # LBH589, Adoq Bioscience), 25 ng/mL bryostatin (Sigma; Cat # B7431), 50 ng/mL PMA (Sigma; Cat #, P8139), and 1 μM SN-38 (Selleckchem; Cat # S4908). DMSO control received fresh media and DMSO and unstimulated control received fresh media only.

### Analysis of latency reactivation in an Ex Vivo model of latent cells derived from primary CD4^+^ T-cells:

To test feasibility of applying smRNA-FISH + IF on a primary CD4^+^ T-cell latency model, we used the Laura Martin method of generating primary latent cells as described^59^. PBMCs were obtained from HIV-1 naive donors, CD4^+^ T-cells were isolated and activated under conditions that block polarization (cultured T_CM_ cells). On day 7 cells were infected with HIV-1_NL4-3_ and allowed to crowd in order to spread the infection. On day 13 the infected cells were un-crowded, treated with IL-2 and cART in order for the infection to become dormant and to allow for the CD4^+^ T cells to enter latency without further spread of the virus. On day 17, the latent CD4^+^ cells were activated with α-CD3/α-CD28 antibodies and IL-2 (activated cells) or treated with IL-2 alone (uninduced controls) for 6, 12, 24, 48 and 72 h, in the presence of continuous cART. The reactivated latent CD4^+^ cells were fixed after latency reversal and analyzed by RNA-FISH + IF.

### Analysis of RNA isolated from primary latent cells from aviremic patients

Aviremic HIV-1 infected patients on cART were recruited for phlebotomy according to an approved and active institutional review board (IRB) protocol at the University of Utah (IRB_0058246) as described previously^44^. Inclusion criteria for this study required viral suppression (less than 50 HIV-1 RNA copies/mL) for a minimum of six months, ART initiation during chronic HIV-1 infection (greater than six months since seroconversion), and compliance with a stable ART regimen for a minimum of twelve months per participant and provider report. *Ex vivo* cell culture assays with resting CD4^+^ T cells from HIV-1-infected aviremic participants were performed as previously described^44,60^. Quantitative PCR (qPCR) to detect HIV-1 mRNA transcripts was performed on cell-associated RNA (caRNA) isolated from cultured cells as described previously^44,60^. RNA isolation and purification were performed using Trizol RNA isolation method (www.invitrogen.com) according to manufacturer’s instructions.

#### RNA-FISH + IF analysis of latent T-cell lines:

The latent T-cells were treated with or without specified drug for indicated duration. The treated/untreated cells were washed three times with 1x PBSM (1x PBS with 5 mM of MgCl_2_) followed by fixation with 4% paraformaldehyde for 30 min. After fixing, these cells were plated onto the wells of either two- or four-chambered slides by dropping and spreading. The cells were first washed with 1x PBSM followed by 10 min. incubation with 0.1M Glycine/PBSM at RT (quenching). After quenching, the cells were washed with 1x PBSM two times for 10 min. each. Cells were then permeabilized using 0.1% TritonX100 in 1x PBSM (1 ml per well in two-welled chambers) for 10 min. Cells were washed two times for 10 min. each with 1x PBSM. After washing, cells were incubated with pre-hybridization buffer (10% deionized formamide in 2x SSC) for 30 min. at RT. HIV-1 gag RNA probe mixture (250 nM final concentration, Biosearch Technologies, Custom Stellaris Probes, Supplementary Table ST1) was added to hybridization buffer [10% deionized formamide (Cat # 75-12-7; Acros Organics), 1 mg/mL competitor *E. coli* tRNA MRE 600 (Cat # 10109541001; Millipore Sigma), 10% dextran sulfate (Cat # D61001, Sigma), 0.2 mg/mL UltraPure BSA (Cat # AM2616; Ambion^™^), 2x Sodium Chloride-Sodium Citrate (SSC) buffer (Cat # R019, G-Biosciences), 2 mM Ribonucleoside Vanadyl Complex (Cat # S1402S; New England Biolabs), 10 U/mL SUPERase•In and 18.6% UltraPure DEPC treated water (Invitrogen; Cat # 750023)]. 500 or 250 μl of hybridization buffer containing HIV-gag RNA probe were added to each well of two- or four- chambered slides respectively and incubated overnight at 37°C incubator under humid conditions. When combining RNA-FISH with IF, the primary antibodies, α-goat p24 antibody (1:500 dilution, a kind gift from Dr. David Ott, NCI) or α-cMYC (9E10) antibody (1:500 dilution, Santa Cruz Biotechnology, Inc; Cat # sc-40) were added to hybridization buffer. Post incubation, the next day, samples were washed with prehybridization buffer three times. If combining with IF, after the three washes, the slides were incubated two times for 20 min. each with the secondary Ab (1:1000 dilutions of α-goat, AlexaFluor 647 and IR-Dye680RD goat-α-mouse antibody, when using α-p24 and α-cMYC (9E10) primary antibodies respectively) in the pre-hybridization buffer (Jackson ImmunoResearch Inc.; Cat # 705-606-147). Following incubation with the secondary antibody, samples were rinsed 3x with 2x SSC and incubated further for 2 hours with 2x SSC buffer. DAPI staining (1.0 μg/ml DAPI in 2x SSC) was carried out for 2 min at RT. After DAPI staining the cells were washed for 5 min. with 2xSSC and mounted using the ProLong Gold antifade mountant (Cat # P10144, Thermo Scientific) and kept at RT in the dark. Next day, the slides were examined under the microscope. If the slides were stored at 4C before examination, the slides were brought to RT followed by microscopy examination.

### smRNA-FISH + IF analysis of primary cells

RNA-FISH and IF analysis of reactivated primary cells was essentially similar to that described for the cell lines, with additional modifications. The cells were washed three times with PBS, fixed for 20 mins in 4% paraformaldehyde and plated onto poly-L-lysine treated chamber-well slides (Cat # 354108, BD Biosciences). Cells were then blocked at RT for 5 min with 0.1% sodium borohydride (in H_2_O, Cat # 452883, Sigma), permeabilized at RT for 2 min. in 0.5% TritonX100, resuspended in PBS. Samples were then washed 3x with ice cold PBS and treated with prehybridization buffer consisting of 2x SSC with 30% formamide and 2 mM RVC (Cat # S1402S; New England Biolabs) and incubated with hybridization buffer containing RNA probe and primary antibody overnight at 37°C in a large plastic petridish, with lids of 15 mL Falcon tube filled with prehybridization buffer and H_2_O-soaked paper wipes kept on the side to maintain moisture levels. The container was sealed using parafilm. The next day the samples were washed 3x for 10 min at 37°C with prehybridization buffer and then incubated 2x for 20 min at 37 °C with prehybridization buffer containing anti-goat-Cy5 antibody. Following incubation with the secondary antibody samples were washed 3x with 2x SSC at RT and fixed again with 4% paraformaldehyde for 30 min. Cells were stained with 0.5 μg/μL DAPI in 2x SSC for 1 min at RT and washed for 5 min with 2x SSC. Coverslips were then mounted on to slides using ProLong Gold antifade mountant (Cat # P10144, Thermo Scientific).

#### HSHRS analysis:

Fluorescent scanning of the slides was done in two stages. First, slide scanning of the entire slides were obtained at 20x using PANNORAMIC 250 Flash III Slide Scanner (RRID:SCR_022184). Automatic focusing (default factory settings) was used for scanning the whole slide. Fluorescence imaging was performed using specific fluorescence filters - DAPI, Cy5-Q, and TRITC-Dendra. Pannoramic scanner software was used for image acquisition and scanned slides were visualized using CaseViewer 2.4 (64-bit version; RRID:SCR_017654). For setting the parameters for image acquisition, mock control was used to set the background level of the fluorescence in the test images. After scanning, the same slides were subjected to single cell imaging.

#### Single molecule image acquisition and analysis:

Cells were imaged using an upright, wide-field Olympus BX-63 Microscope equipped with a SuperApochromatic 60×/1.35 NA Olympus Objective (UPLSAPO60XO), a SOLA light engine (Lumencor), an ORCA-R2 Digital Interline CCD Camera (C10600-10B; Hamamatsu), and zero-pixel shift filter sets: DAPI-5060C-Zero, Cy3-4040C-Zero (for Quasar 570 detection), and Cy5-4040C-Zero (for Quasar 670 detection) from Semrock, as described^61^. The resulting image pixel dimension was 107.5 nm, and the z-step size (along the optical axis) used for all optical sectioning acquisition was 200 nm. Metamorph software (Molecular Devices; RRID:SCR_002368) was used for controlling microscope automation and image acquisition. Exposure times and gain settings were variable for CY5, CY3, and DAPI. Laser was at 100% for Cy3 and Cy5 and at 12% for DAPI. Typical settings are as follows. Exposure times for CY5, CY3, and DAPI were 25 ms, 200 ms, and 10 ms. Gain for CY5, CY3, and DAPI were 2, 20, and 2, respectively. Images were analyzed using ImageJ and/or Fiji software (RRIDs:SCR_003070 and SCR_002285)^62^.

#### Quantification of mature and nascent transcripts using FISH-quant Analysis:

FISH-quant analysis was performed in MATLAB (R2017b; RRID:SCR_001622) using FISH-quant (v3a) software as described by Mueller et al^26^. The settings in these experiments were adjusted to pixel size (xy) 107.5, Pixel size 200, RF 1.518, aperture 1.4, emission 566, excitation 548 to fit experimental parameters. To generate the average mature transcript image, the size of region around the center [XY] was 9, the size of the region around the center [Z] was 7, the background was not subtracted for each point, and normal sampling was used. Greater than 1000 spots were averaged for each analysis.

#### Single molecule statistical analyses:

Statistical testing involving generation of Fano Factor, variance, and 95% confidence intervals was completed in Rstudio (version 0.98.1103; RRID:SCR_000432), using bootstrapping with 10,000 simulations to generate confidence intervals using R (version 3.1.2; RRID:SCR_001905)^63^.

### Graph generation

Histograms, and Fano Factor/variance graphs were generated in Rstudio. All other graphs were created using GraphPad Prism 9.5.1.

### Quantitative RT-PCR of reactivated HIV-1 RNA to compare to that of FISH-Quant analysis:

ACH2 cells were activated using 50ng/ml of PMA and cell lysate were collected 24 h post activation. RNA was extracted from the cells using the RNeasy kit (Qiagen; Cat # 74104) and quantified via nanodrop. 2 μg total RNA was reverse transcribed, and the cDNA was subjected to real time PCR using HIV-1 late reverse transcription primers, forward (5’-TGTGTGCCCGTCTGTTGTGT-3’) and reverse (5’-GAGTCCTGCGTCGAGAGAGC-3’), to detect HIV-1 transcripts. An equal amount of DNaseI-treated RNA that was not reverse transcribed was used as a negative (no RT) control. Real Time-PCR was performed using and ABI7700 and analyzed using SDS2.1 software. PCR conditions were as follows: initial annealing at 50 °C for 2 min, initial denaturation at 95 °C for 10 min., followed by 40 cycles of 95 °C for 10 sec., 60 °C for 20 sec., 72 °C for 30 sec. A standard curve of the Cesium Chloride prepped pNL4-3 DNA was generated and used to estimate the number of molecules/reaction. Number of viral transcripts/cells in the PCR reaction was estimated by dividing the number of molecules/reactions with number of cells/reaction.

### Single cell RNA-seq analysis

scRNA-seq was performed using the 10x Genomics Chromium platform. Single-cell mRNA libraries were built using the Chromium Next GEM Single Cell 3’ Library Construction V3.1 Kit, and the libraries are sequenced using Illumina NextSeq 500 (Illumina, San Diego, CA, USA) to obtain pair-end sequencing of 26 bp (read1) × 98 bp (read2) and a single index 8bp in length. CellRanger Single Cell Analysis Pipelines (10x Genomics, Pleasanton, CA, USA) was used to align and count to the reference genome and transcripts. For this purpose, the human genome (GRCh38) and HIV-1_HXB2_ complete genome (GenBank K03455.1) were merged for the alignment. The scRNA-seq data was analyzed using an R package, Seurat^64^. Standard QC was performed, removing cells expressing < 200 genes and genes expressed in < 3 cells in each sample. For each sample, the number of cells, the distribution of the numbers of genes per cell, the number of unique molecule identifiers and the percentage of these mapping to mitochondria were assessed. These and other metrics were used to remove outliers. Dimensionality reduction was performed by principal component analysis (PCA) and highly variable genes were identified separately for each sample, to verify that the patterns are comparable. We then calculated a Euclidean distance matrix based on principal components (PCs), and performed iterative graph-based clustering as implemented in Seurat to classify each cell-to-cell subtype clusters^64^. We classified cell subtype clusters based on HIV gene expression status to identify differentially expressed genes (DEGs) between HIV gene-positive and negative cells in each sample. DEGs should be at least 25% of cells in the cluster expressing the gene, at least two-fold differences between the group and statistically significant. The significance of changes was tested with a hurdle model tailored to scRNA-seq data, which was implemented in the Seurat’s *FindMarker* function as “MAST”, and a false discovery rate (FDR) adjusted p value threshold of 0.05 was applied.

#### qRT-PCR analysis to validate the DEGs:

Total RNA was extracted from the respective latent cell lines using the RNeasy Plus Mini Kit (QIAGEN; Cat # 74134), and its concentration and purity were assessed using Nanodrop. 2 μg of total RNA from each sample were subjected to cDNA synthesis using the iScript^™^ Advanced cDNA Synthesis Kit (BIO-RAD; Cat # 1725037) following the manufacturer’s instructions. The qRT-PCR reaction mixture contained 5 μL SsoAdvanced^™^ Universal Inhibitor-Tolerant SYBR^®^ Green Supermix (BIO-RAD; Cat # 1725016), 0.5 μL gene-specific forward or reversed primer (See the Table of primers below), 4 μL nuclease-free water (Sigma; Cat # W4502-50ML), and 0.5 μL cDNA (1:10 diluted from the above steps). An equal amount of DNaseI-treated RNA was used as a negative (no RT) control, whereas the *GAPDH* gene served as an internal control. The qRT-PCR was performed on Applied Biosystems ViiA 7 system using the 384-well plate and analyzed using QuantStudio^™^ Real-Time PCR Software (Applied Biosystems). The initial denaturation of qRT-PCR was at 95°C for 10 min followed by 40 cycles including strand separation at 95°C for 10 s, annealing at 60°C for 20 s, and extension at 72°C for 30 sec.

## Figures and Tables

**Table:** Table of Primers used in qRT-PCR analysis to validate the DEGs.

Primer Name	Primer Sequences (5’ to 3’)	Product Size (bp)
h_c-Myc_1913_Fw	CCTGGTGCTCCATGAGGAGAC	128 bp
h_c-Myc_2040_Rv	CAGACTCTGACCTTTTGCCAGG	
h-GAPDH_Fw_920	GTCTCCTCTGACTTCAACAGCG	131 bp
h-GAPDH_Rv_1050	ACCACCCTGTTGCTGTAGCCAA	
RGS16_Fw_269	AAACACAGCAAAGAGAATAGAAACT	249 bp
RGS16_Rv_517	CTCTTTAGGGGCCTCACTGC	
NR4A2_Fw_1086	GAGTCTGATCAGTGCCCTCG	155 bp
NR4A2_Rv_1240	TGGAGCCAGTCAGGAGATCA	
EGR3_Fw_28	CCGGTGACCATGAGCAGTTT	158 bp
EGR3_Rv_185	TCGTTGGTCAGACCGATGTC	
NR4A3_Fw_2	TGCATGACTCAATCAGATTTGGA	160 bp
NR4A3_Rv_161	CCAAGGTCCATGGTCAGCTT	
NR4A1_Fw_1489	CTTGTCGATGTCCCTGCCTT	187 bp
NR4A1_Rv_1675	GTTTGCCCAACAGACGTGAC	

## Data Availability

All single cell RNA-seq data has been submitted to GEO under the accession number GSE241207. All other data will be provided by the corresponding author upon request after publication.
